# A Dual Protection System for Heterostructured 3D CNT/CoSe_2_/C as High Areal Capacity Anode for Sodium Storage

**DOI:** 10.1002/advs.201902907

**Published:** 2020-01-21

**Authors:** Muhammad Yousaf, Yijun Chen, Hassina Tabassum, Zhipeng Wang, Yunsong Wang, Adeel Y. Abid, Asif Mahmood, Nasir Mahmood, Shaojun Guo, Ray P. S. Han, Peng Gao

**Affiliations:** ^1^ Department of Material Science and Engineering Peking University Beijing 100871 China; ^2^ International Center for Quantum Materials and Electron Microscopy Laboratory School of Physics Peking University Beijing 100871 China; ^3^ School of Chemical and Biomolecular Engineering The University of Sydney 2006 Sydney Australia; ^4^ School of Engineering RMIT University 124 La Trobe Street Melbourne Victoria 3001 Australia

**Keywords:** 3D electrodes, CNT/CoSe_2_/C, dual conductive network, high areal capacity, sodium‐ion batteries

## Abstract

3D electrode design is normally opted for multiple advantages, however, instability/detachment of active material causes the pulverization and degradation of the structure, and ultimately poor cyclic stability. Here, a dually protected, highly compressible, and freestanding anode is presented for sodium‐ion batteries, where 3D carbon nanotube (CNT) sponge is decorated with homogeneously dispersed CoSe_2_ nanoparticles (NPs) which are protected under carbon overcoat (CNT/CoSe_2_/C). The 3D CNT sponge delivers enough space for high mass loading while providing high mechanical strength and faster conduction pathway among the NPs. The outer amorphous carbon overcoat controls the formation of solid electrolyte interphase film by avoiding direct contact of CoSe_2_ with electrolyte, accommodates large volume changes, and ultimately enhances the overall conductivity of cell and assists in transmitting electron to an external circuit. Moreover, the hybrid can be densified up to 11‐fold without affecting its microstructure that results in ultrahigh areal mass loading of 17.4 mg cm^−2^ and an areal capacity of 7.03 mAh cm^−2^ along with a high gravimetric capacity of 531 mAh g^−1^ at 100 mA g^−1^. Thus, compact and smart devices can be realized by this new electrode design for heavy‐duty commercial applications.

## Graphical abstract

A 3D and dually protected electrode design (carbon nanotube (CNT)/CoSe_2_/C) is presented in which a CNT sponge is decorated with CoSe_2_ nanoparticles and then externally protected with carbon layers. The representative 3D CNT/CoSe_2_/C heterostructure has the ability to be densified without influencing its microstructure that results in high areal capacity along with high gravimetric capacity for sodium‐ion batteries.

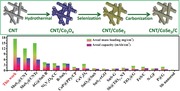

Owing to even distribution and a large abundance of sodium (Na), sodium‐ion battery (SIB), the most promising alternative to lithium‐ion battery (LIB), has received considerable attention in the last few years.^1^, ^2^, ^3^, ^4^ However, the larger radius of Na^+^ (0.102 nm) compared with Li^+^ (0.076 nm) sluggish its kinetics and collapse the structure of electrode materials during insertion/de‐insertion processes, therefore, better host materials need to be found that are suitable for SIBs.^5^, ^6^ Transition metal diselenides, a class of transition metal chalcogenides (TMC) based material, are very promising candidates as an anode for SIB due to their higher theoretical capacity (≈494.36 mAh g^−1^ for CoSe_2_) than graphite (≈35 mAh g^−1^)^7^, ^8^ and low synthesis cost.^9^, ^10^ Nevertheless, severe volume changes during sodiation/desodiation process, slow transport of charges, and diffusion of Na^+^ along with high intrinsic resistance resulted in capacity fading and hindered the practical application of transition metal diselenides.^9^, ^11^ Until now, a lot of efforts have been devoted to overcome these issues by synthesizing transition metal diselenides based nanostructures with various shapes, morphologies, and particle sizes.^10^, ^11^, ^12^

Although creating voids, hollow, or porous nanostructures have improved the gravimetric capacity due to their ability to accommodate the volume expansion, shorten the diffusion path for charge transportation, optimized the electrode and electrolyte contact area, etc.^13^, ^14^ However, downsizing the materials at the nanoscale is not always in favor of rechargeable batteries for practical applications.^15^ Because the nanomaterials possess low tap density that results in low areal mass loading (<1 mg cm^−2^), consequently lowering the areal and volumetric capacities which are very important metrics in practical applications.^16^, ^17^ Traditional high areal mass loadings of active material bring high electrode thicknesses which severely limits the in‐depth accessibility of active materials ultimately fading the reversible capacity.^15^, ^18^ Moreover, mixing of the binder is required in traditional electrode preparation which decreases the overall conductivity of the electrode, increase cell resistance, and more profound to the volume variations. While these volume changes during repeated charging/discharging produce cracks and cause pulverization of electrode, hence bringing poor electrical contact of active material with the current collector or even detaching of the active materials from the current collector.^19^, ^20^ To resolve these issues various electrode architecture designs have been proposed using copper as current collector.^21^ But, copper‐based current collector electrodes may increase the overall volume and decrease the energy density of the device. To resolve this issue 3D, freestanding electrode configurations are very attractive, where the active materials are directly grown over 3D conductive scaffold alleviating the need of any dead additives.^22^ Among various 3D conductive scaffolds, 3D carbon nanotube (CNT) sponges have found a lot of attention lately due to their ultrahigh porosity, conductivity, and well‐interconnected 3D networks that not only provide high mass flow but also allow high mass loading in minimal area.^23^, ^24^, ^25^, ^26^ However, several challenges limit the application of 3D CNT sponge as conductive host; for instance: (i) the selected material should be compatible with the substrate (CNT sponge) and should have enough adsorption energy between the active material and substrate surface to guarantee the tight bond.^27^ (ii) The solubility of two precursors (i.e., transition metal precursor and Se precursor) together is another big issue where poor solubility results in suspension/precipitation instead of the clear transparent solution. Poor solubility affects efficient infiltration of the precursor into the porous host (iii) inability of current approaches to load high amounts of active materials, (iv) detaching of active materials from CNT surface, and (v) poor control over direct electrode/electrolyte contact, and (vi) inhomogenous solid electrolyte interphase (SEI) film formation.

In this work, a novel dually protected electrode design was presented by fabricating CNT/CoSe_2_/C as an anode for SIB. The CoSe_2_ nanoparticles (NPs) are decorated on each individual CNT which comprised a 3D network by simply two‐step hydrothermal processes and then externally protected with amorphous carbon layers through a carbonization process. This unique composite greatly improves the Na^+^ storage by harnessing the unique morphological and electronic features and surface properties. In particular, the highly conductive and compressible 3D CNT sponge provides multichannel and continuous pathways for ionic and electronic conduction, enough space, and porosity for high mass loading of active CoSe_2_ and electrolyte infiltration as well as accommodate the volume expansion upon cycling. In contrast to conventional 3D electrodes, the external dual protection by carbon layers on individual CNT through the 3D matrix not only increases the overall conductivity but also buffers the volume expansion and controlled the SEI film thickness by preventing active material from direct contact with corrosive surrounding. By harnessing all the features, our hybrid is used as a freestanding electrode for SIBs (i.e., without dead elements such as binders, conductive additives, and current collector) and its fabrication is simpler and lighter. It delivers a high gravimetric capacity of 531 mAh g^−1^ at 100 mA g^−1^ after 100 cycles at high mass loading of 17.4 mg cm^−2^ which corresponds to the exceptional areal and volumetric capacity of >7 mAh cm^−2^ and 185.2 mAh cm^−3^, respectively. Hence, by extending such a unique designing to other transition metal chalcogenides will take the electrochemical energy storage devices to the doorstep of commercial installations.

The carbon‐coated 3D CNT/CoSe_2_ hybrid was obtained via three steps as shown in **Figure** **1**a; initially, in situ Co_3_O_4_ NPs were uniformly decorated in a CNT sponge by a hydrothermal process, then, selenization of Co_3_O_4_ into crystalline CoSe_2_, resulting in a 3D CoSe_2_@CNT hybrid without disturbing the morphology. The NaBH_4_ was used to reduce the Se atoms into Se^−2^ anions and Co_3_O_4_ into Co^+2^ cations, which then reacted at 180 °C to form CoSe_2_.^28^, ^29^, ^30^, ^31^ Finally, every individual CNT/CoSe_2_ hybrid structure was coated with a carbon shell by carbonization using glucose at high temperature under a neutral atmosphere. During the cell assembly various hybrid sponges with an initial thickness *h*_i_ were compressed into dense framework with a final thickness *h*_c_ and directly used as freestanding anodes in the coin‐type cell (Figure 1b). The areal mass loading (*M*_a_) or density (*d*) of as‐obtained electrode was determined by *M*_a_ = *dV*/*A* = d*h*, where *d*, *V*, *A*, and *h* are the density, volume, base area, and thickness of the hybrid sponge, respectively. This indicates that the hybrid sponge having same percentage of active mass loading (CoSe_2_) may proportionally increase the *d* and *M*_a_ by simply choosing the CNT framework with larger *h*_i_ and compressing it to fitting *h*_c_ into coin cell. In this way, a densified framework was achieved and so enhanced the density, areal mass loading, and areal capacity.

**Figure 1:**
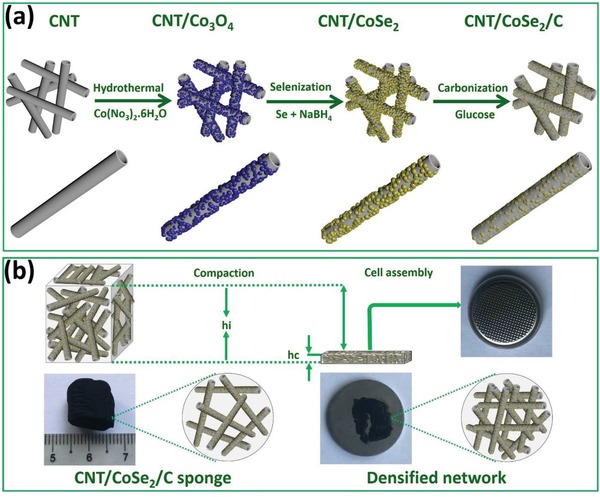
a) Schematic illustration for the fabrication process of CNT/CoSe_2_/C sponge. b) Photographs of CNT/CoSe_2_/C sponge before and after compaction during cell assembly with description of initial (*h*_i_) and final (*h*_c_) thicknesses.

Figure S1 (Supporting Information) shows that the CNTs have a smooth surface with interconnected nanotubes networks as an ideal surface to decorate the active materials. Importantly, it is found that the decoration of Co_3_O_4_ NPs did not bring any change in the 3D network of CNTs, however, rough surface confirms the formation of CNTs/Co_3_O_4_ core–shell structure (**Figure** **2**a). The in situ grafting of Co_3_O_4_ NPs on the surface of CNT ensures enhanced contact between them. Upon conversion of Co_3_O_4_ into CoSe_2_ NPs on CNTs surface (Figure 2b) and coating of the carbon shell on the CNT/CoSe_2_ hybrid (Figure 2c), the 3D structural integrity is well‐maintained pointing the high strength of 3D framework and carbon shell which has no effect on the morphology. Our hybrid morphology is unique and different from previously reported CNT/CoSe_2_ composites, where a simple combination of CNT and CoSe_2_ was obtained. The previous composites are in powder form and may aggregate during long electrochemical cycling.^32^, ^33^ However, in our hybrid, a core–double shell structure like morphology was obtained, which is homogenous in its nature and unique from reported literature. Besides our glucose‐derived carbon shell was homogenously coated over the entire core–shell (CNT/CoSe_2_) structure that has strong interaction with active material and beneficial for long cycling, while its thickness kept as low which allows faster ionic flow between electrode and electrolyte.^32^, ^33^ The transmission electron microscopy (TEM) analysis is used to determine the size of NPs (both Co_3_O_4_ and CoSe_2_), the diameter of nanotube in each hybrid, and thickness of carbon coating. Figure 2d,g reveals that fine Co_3_O_4_ NPs having a size of ≈8 nm are well‐adhered to the CNTs surface which are about 40 nm in diameter. The analysis of CNT/CoSe_2_ revealed up to ≈25 nm increase in particle size due to the ripening of NPs under hydrothermal/calcination reactions as shown in Figure 2e,h. Figure 2f,i are showing the uniform coating of carbon on the surface of a hybrid with an average thickness of ≈4.0 nm. This carbon overcoat is uniformly covering every CoSe_2_ NP forming an intertwined structure in which CoSe_2_ NPs are tightly bound with 3D CNT networks. The new physical properties (such as high conductivity and mechanical strength) and synergistic effect between CoSe_2_ and dual conductive network can be benefitted from no or low volume expansion, limited and stable SEI film, enhanced capacity, and structural integrity upon cycling. To verify the conversion of Co_3_O_4_ to CoSe_2_, high‐resolution TEM (HRTEM) images of CNT/CoSe_2_/C were obtained that represent the lattice spacing of ≈0.30 nm correspond to the (011) plane of CoSe_2_ (**Figure** **3**a). The selected area electron diffraction (SAED) pattern of CNT/CoSe_2_/C is given in Figure 3b, which is well matched with the X‐ray diffraction (XRD) measurements (Figure 3c). XRD patterns were noted at each synthesis process and are presented in Figure S2 (Supporting Information) and Figure 3c. The XRD spectrum of Co_3_O_4_ is well‐matched with the cubic phase (JCPDS No. 42‐1467) showing highly crystalline growth of NPs (Figure S2, Supporting Information), which upon selenization yielded pure CoSe_2_ in hexagonal phase (JCPDS No. 53‐0449) as shown in Figure 3c. However, a reduced peak intensity for CNT/CoSe_2_/C hybrid further confirms carbon coating on the CoSe_2_ decorated CNTs (Figure 3c). Figure S3 (Supporting Information) displays an energy‐dispersive X‐ray (EDX) mapping recorded with the help of scanning electron microscopy (SEM) for carbon, cobalt, and selenium where carbon concentrated at the core, and cobalt and selenium uniformly dispersed along the CNTs. The high‐angle annular dark‐field scanning TEM (HAADF‐STEM) and corresponding EDX mappings are given in Figure 3d. An intense signal of carbon is observed in the middle, whereas the cobalt and selenium are concentrated over the CNT core across the shell. A weak signal of carbon is also observed across the CoSe_2_ NPs because of the thinner carbon overcoat.

**Figure 2:**
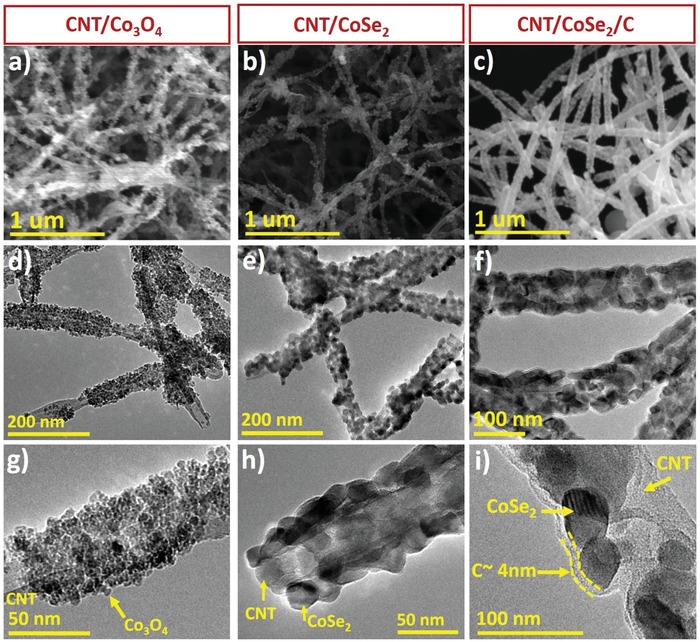
Morphological characterizations of hybrid sponges: a–c) SEM images of CNT/Co_3_O_4_, CNT/CoSe_2_, and CNT/CoSe_2_/C, respectively. TEM images of d,g) CNT/Co_3_O_4_, e,h) CNT/CoSe_2_, and f,i) CNT/CoSe_2_/C showing diameter of outer carbon layer of ≈4.0 nm.

**Figure 3:**
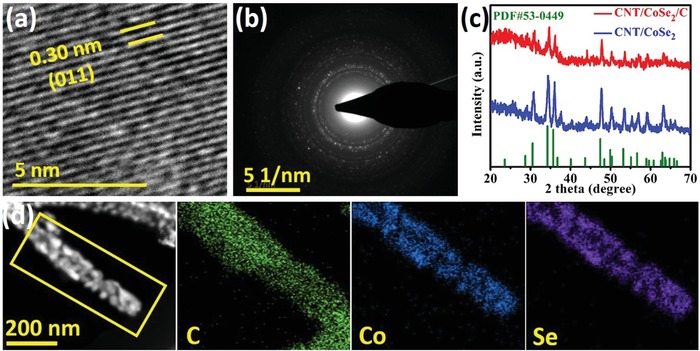
Crystal structure and elemental measurements of hybrids: a) HRTEM of CNT/CoSe_2_/C, b) XRD patterns of CNT/CoSe_2_ and CNT/CoSe_2_/C, c) SAED pattern of CoSe_2_, and d) elemental mappings of C, Co, and Se in CNT/CoSe_2_/C hybrid showing homogenous distribution of Co and Se along CNT.

**Figure** **4**a displays the Raman spectrum of CNT, CNT/CoSe_2_, and CNT/CoSe_2_/C. In addition to the G‐band (1583 cm^−1^) and D‐band (1348 cm^−1^) coming from CNTs, the sharp peak at 188 cm^−1^ in both CNT/CoSe_2_ and CNT/CoSe_2_/C corresponds to the Se—Se stretching mode of cubic CoSe_2_,^34^, ^35^, ^36^ whereas a small peak at about 672 cm^−1^ is associated with A_1g_ mode of CoSe_2_.^37^ The valence states of different element in CNT/CoSe_2_/C were evaluated using X‐ray photoelectron spectroscopy (XPS) technique. The survey spectrum shows the peaks of Co, Se, C, and O indicating high purity of hybrid (Figure S4, Supporting Information). The high‐resolution spectrum of Co 2p region indicates binding energies of Co 2p_3/2_ and Co 2p_1/2_ located at 778.4 and 793.45 eV, respectively, confirming the Co^2+^ cations in CoSe_2_ (Figure 4b).^34^, ^35^, ^36^ Moreover, two peaks located at 780.83 and 796.2 eV correspond to Co—O (a thin oxide layer on the surface) and one satellite peak at 801.13 eV was also observed in Co 2p region (Figure 4b).^38^, ^39^ Figure 4c shows the doublet of Se 3d, the peaks at 54.12 and 54.98 eV are related to Se 3d_5/2_ and Se 3d_1/2_, respectively, well‐consistent with metal selenide bond in CoSe_2_, while the peaks at 59.12 and 60.82 eV arise from Co 3p_3/2_ and Co 3p_1/2_ of Co^2+^.^40^, ^41^ The C 1s spectrum presented four subpeaks at 284.35, 284.77, 285.71, and 289.2 eV corresponding to C—C, C=C, C—O, and O—C=O bonds, respectively, originating from CNTs core and amorphous carbon shell (Figure 4d).^42^

**Figure 4:**
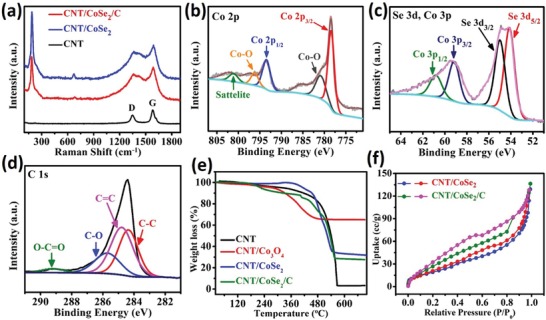
Structural characterizations of hybrid sponges: a) Raman spectra of CNT, CNT/CoSe_2_, and CNT/CoSe_2_/C, respectively. b–d) High‐resolution XPS spectra of Co 2p, Se 3d, and C 1s, respectively. e) TGA analysis of various sponges carried out in air for various synthesized products. f) BET surface area of CNT/CoSe_2_ and CNT/CoSe_2_/C, respectively.

The thermogravimetric analysis (TGA) was carried out in the air to determine the exact amount of carbon in each composite at various stages (the procedure to calculate the carbon content in various hybrid sponges is provided in the Supporting Information). In CNT/Co_3_O_4_ hybrid, a major weight loss occurred at between 300 and 500 °C, while for CNT/CoSe_2_ hybrid, first, the weight increased till 400 °C due to oxidation of Se into SeO_2_ and then decreased due to the removal of CNTs through oxidation (Figure 4e). The final products mainly consisted of Co_3_O_4_ and a small amount of residual Fe catalyst used to grow CNTs sponge in all composites. The calculated amount of carbon in CNT/Co_3_O_4_, CNT/CoSe_2_, and CNT/CoSe_2_/C is ≈35.07%, ≈11.84%, and 25.6%, respectively. The content of amorphous carbon coating in CNT/CoSe_2_/C hybrid is calculated to be 17.3%. Moreover, Brunauer–Emmett–Teller (BET) surface area and pore size distribution for various hybrids are shown in Figure 4f and Figure S5 (Supporting Information), respectively, and corresponding values are presented in Table S1 (Supporting Information). The CNT sponge used here has a higher surface area of 135.98 m^2^ g^−1^.^43^ The lower surface area in case of CNT/CoSe_2_ (50.24 m^2^ g^−1^) is due to the homogenous coating of CoSe_2_ NPs. However, increased surface area in case of CNT/CoSe_2_/C (74.02 m^2^ g^−1^) is attributed to uniform encapsulating and intertwining of amorphous carbon, which is useful for the better contact between the electrolyte and active material, resulting in enhanced pseudocapacitive contribution in Na storage.^44^

The cyclic voltammetry (CV) of the CNT/CoSe_2_/C at a scan rate of 0.30 mV s^−1^ in the voltage range of 0.01–3.00 V was recorded to evaluate its Na^+^ storage capability (**Figure** **5**a). In the first discharge process, one weak and one dominating cathodic peaks were observed at 1.03 and 0.52 V, respectively. The weak peak located at 1.03 V is associated with the intercalation process with the formation of Na*_x_*CoSe_2_ intermediate,^45^ whereas the strong peak at 0.52 V is attributed to the conversion reaction from Na*_x_*CoSe_2_ into metallic Co and Na_2_Se along with decomposition of electrolyte and formation of stable SEI film.^46^, ^47^, ^48^ During the first charge process, a strong oxidation peak at 2.01 V was observed that ascribed to the recovery of CoSe_2_ from the Co metal and Na_2_Se.^46^, ^47^, ^48^ In the following cycles, two reduction peaks located at ≈0.43 and ≈0.97 V and one oxidation peak at ≈2.02 V were observed and almost remain steady and overlapped that reveals the stability and reversibility of the hybrid for the sodiation and desodiation processes (Figure 5a). The galvanostatic discharge/charge voltage profile curves of CNT/CoSe_2_ and CNT/CoSe_2_/C at a current density of 100 mA g^−1^ are displayed in Figure 5b. In the first discharge cycle, long voltage plateaus at 0.9 and 0.5 V were observed with a gradual voltage drop and consistent with CV findings. The CNT/CoSe_2_ and CNT/CoSe_2_/C showed initial discharge and charge capacities (normalized to active mass of the electrodes) of 857 and 535, 952, and 544 mAh g^−1^, respectively. Whereas normalized to the total mass of electrodes, the corresponding discharge and charge capacities would be 755 and 471, 708, and 404 mAh g^−1^, respectively. The initial Coulombic efficiency of CNT/CoSe_2_ and CNT/CoSe_2_/C was 62.47% and 57.09%, respectively. The slightly low Coulombic efficiency for the initial cycles in CNT/CoSe_2_/C is due to the formation of SEI film for thermodynamic stability of the cell and amorphous carbon coating which leads to a large surface area that causes irreversible loss of electrolyte for the first cycle.^49^, ^50^ However, a smaller difference between first and second charge capacities of CNT/CoSe_2_/C (5 mAh g^−1^) than CNT/CoSe_2_ (37 mAh g^−1^) and so on for next cycles (Table S2, Supporting Information) was observed, highlighting the advantages of protective carbon coating.

**Figure 5:**
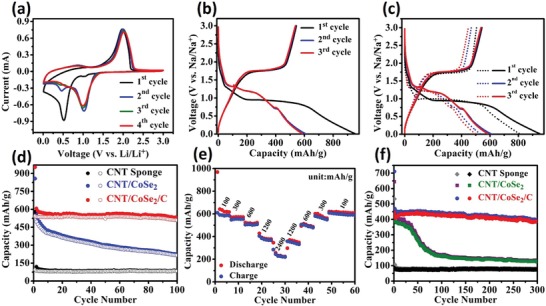
Electrochemical storage behaviors of various electrodes in SIBs: a) CV curves at a scan rate of 0.2 mV s^−1^ in CNT/CoSe_2_/C. b) Charge/discharge voltage profiles of CNT/CoSe_2_/C at 100 mA g^−1^. c) Comparison of charge/discharge voltage profiles between CNT/CoSe_2_ and CNT/CoSe_2_/C; dotted curves are for CNT/CoSe_2_ and solid curves are for CNT/CoSe_2_/C. d) Comparison of cycling performance among CNT/CoSe_2_,CNT/CoSe_2_/C and CNT sponge, respectively. e) Rate capability of CNT/CoSe_2_/C. f) Cyclic stability of pure CNT sponge, CNT/CoSe_2_, and CNT/CoSe_2_/C at 500 mA g^−1^.

The effect of carbon coating on the electrochemical Na^+^ storage is further evaluated by a comparative study of cycling behaviors. It can be concluded that discharge capacity almost remains stable with increasing the cycle number in the CNT/CoSe_2_/C electrode, that is, 531 mAh g^−1^ at 100 mA g^−1^ after 100 cycles (Figure 5d). However, the specific capacity of CNT/CoSe_2_ electrode gradually decreased with cycle number and retained only 223 mAh g^−1^ after 100 cycles, highlighting the significance of carbon overcoat in improving cyclic stability and control over SEI film with ongoing cycles. Theoretically, the gravimetric capacity (*C*_g_) of the CNT/CoSe_2_ and CNT/CoSe_2_/C electrodes can be calculated from the capacity of the pure CNT sponge (87.1 mAh g^−1^) at 100 mA g^−1^ in this work (Figure 5d), theoretical capacity of CoSe_2_ (494.34 mAh g^−1^), and the percentage loading of CoSe_2_ in CNT/CoSe_2_ (88.16 wt%) and CNT/CoSe_2_/C (74.4 wt%) using the following formula

(1) $$\begin{array}{l} C_{\text{g}}\;\text{of}\;{\text{CNT/CoSe}}_{2}\;\text{electrode}\\ \;\;\;\;=87.1\times 11.84\%+494.34\times 88.16\%=446.15\;\text{mAh}\;{\text{g}}^{-\text{1}} \end{array}$$

(2) $$\begin{array}{l} C_{\text{g}}\;\text{of}\;{\text{CNT/CoSe}}_{2}\text{/C}\;\text{electrode}\;\\ \;\;\;\;=\;87.1\times 25.6\%+494.34\times 74.4\%=390.05\;\text{mAh}\;{\text{g}}^{-\text{1}} \end{array}$$

The gravimetric capacity obtained in CNT/CoSe_2_/C hybrid after 100 cycles at 100 mA g^−1^ normalized to total mass of electrode is larger (395.1 mAh g^−1^) than theoretical capacity of the electrode (390.05 mAh g^−1^) and this can be attributed to the synergistic effects in the hybrid structure. The rate performances of the CNT/CoSe_2_/C at different current densities ranging from 100 to 2400 mAh g^−1^ are further evaluated as presented in Figure 5e. The CNT/CoSe_2_/C anode exhibits high reversible capacities of 378.5 and 223.6 mAh g^−1^ even at high current rates of 1200 and 2400 mAh g^−1^, respectively. Interestingly, as the currents were reversibly changed from 2400 to 1200, 600, 300, and 100 mA g^−1^, the discharge capacities were fully recovered to 223.6, 348, 486, 560, and 609 mAh g^−1^, respectively. Further, Figure 5f reveals the long cyclic stability of the CNT/CoSe_2_/C at 500 mA g^−1^ and the discharge capacity of 396 mAh g^−1^ is still maintained after 300 cycles. The discharging/charging curves at 500 mA g^−1^ of various cycles are provided in Figure S7a (Supporting Information), which clearly shows similar behavior regardless of number of time electrode is charged or discharged. We have also calculated the Coulombic efficiency of our CNT/CoSe_2_/C sponge. It is low in initial cycles mainly due to amorphous carbon coating due to irreversible Na ions in initial cycles because of formation of SEI film. However, after few cycles the Coulombic efficiency almost remains stable nearly equal to 100% (Figure S7b, Supporting Information) that reveals the high reversibility of our hybrid sponge. These electrochemical results reveal the CNT/CoSe_2_/C electrode has good reversibility and high cyclic stability due to the synergistic effect of multicomponent hybrid. The CNT frameworks provide 3D conductive pathways for efficient charge transport and faster electrolyte penetration due to the intrinsic porous structure of the sponge. The CoSe_2_ provides sufficient active sites for Na^+^ storage, whereas the carbon overcoat not only provides the mechanical robustness for volume expansion during long cycling but also prevents the corrosive surrounding between the active material (CoSe_2_) and electrolyte.

In addition, areal or volumetric capacity is also important battery metrics for real applications like in compact devices and has gained less attention in previous studies for SIBs. Our CNT/CoSe_2_/C sponge can be densified by compaction and using this unique property, the high mass loading and areal capacity can be achieved. The hybrid sponges with various initial thicknesses of 2.1, 2.5, 2.8, 3.3, and 4.2 mm were compacted into coin cell electrodes corresponding to a compression ratio of 6.17, 7.24, 7.74, 9.7, and 11.02, respectively. It indicates that hybrid sponge consists of highly porous microstructure that after a high degree of compaction still, it can maintain desirable porosity (**Figure** **6**a,b) like 11 times reduction in thickness corresponding to the compressive strain of ≈90%. The compressive stress–strain curves of CNT/CoSe_2_/C sponge indicate elastic deformation and good reversibility of the hybrid sponge after 30%, 60%, and 90% compressive strain (Figure 6c). During the compressive unidirectional stress, the base area and side size of the hybrid sponge did not change, showing that close stacking of nanotubes and decrease of inherent porosity of the sponge are responsible for the reduction of thickness.

**Figure 6:**
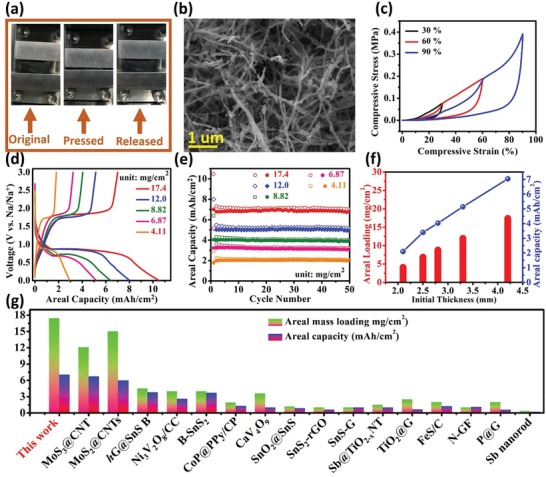
Mechanical and electrochemical tests for CNT/CoSe_2_/C electrodes with various mass loadings: a) Photos of compressible test at original, pressed, and after released states. b) SEM image of compacted CNT/CoSe_2_/C with porous networks. c) Compressive stress–strain curves at different strain = 30%, 60%, and 90%. d,e) The first discharge/charge curves and areal capacity versus cycle number of hybrid sponge anodes with various mass loadings at 100 mA g^−1^, respectively. f) The relationship between areal capacities of hybrid sponges with different areal mass loadings. g) Literature survey of areal performance metrics of some representative SIB anodes.

The mechanism of areal capacity for Na^+^ storage with different mass loading of 4.11, 6.87, 8.82, 12.0, and 17.4 mg cm^−2^ is investigated and displayed in Figure 6d. The curve shapes and voltage plateaus, showing similar charge storage mechanisms like that of the original hybrid, confirm no compositional or structural change occurred after high compression. The cyclic stability of various electrodes with variable areal loading is presented in Figure 6e. The electrode with 17.4 mg cm^−2^ areal loading delivered an initial areal capacity of 10.48 mAh cm^−2^ at 100 mA g^−1^ current density and kept 7.04 mAh cm^−2^ after 50 cycles. Similarly, areal capacities of 5.15, 4.03, 3.4, and 2.1 mAh cm^−2^ were obtained corresponding to areal mass loading of 12.0, 8.82, 6.87, and 4.11 mg cm^−2^, respectively (Figure 6f). The relation among the initial thickness (*h*_i_), areal mass loading (*M*_a_), density (*d*), and areal capacity of electrodes are provided in Figure 6f and Figure S8 (Supporting Information). The highest thickness sponge (4.2 mm) anode resulted in higher density, areal mass loading, and areal capacity (Figure 6f). We can pack the sponges with more thickness into the cells during assembling to further enhance the mass loading and density. But, indiscriminate packing might consequence the capacity fading and poor cyclic stability.^51^ Therefore, the areal capacity of a compressible electrode could be tuned by selecting the appropriate initial thickness to reveal an anticipated mass loading. To the best of our knowledge, this mass loading and areal capacity for SIBs achieved in our CNT/CoSe_2_/C hybrid sponge are much higher compared with reported literature as shown in Figure 6g and presented in **Table** **1**. This enhanced areal capacity is ascribed to the high surface area, porosity, and dense compaction of hybrid sponge, which on compression can load more mass in small area and consequently enhance the areal capacity.

**Table 1:** A survey of areal mass loading and areal capacity of various electrode materials for SIBs

Electrode material	Mass loading [mg cm^−2^]	Areal capacity [mAh cm^−2^]	Ref.
CNT/CoSe_2_/C	17.4	7.04	This work
MoS_3_@CNT	12.1	6.7	52
MoS_2_@CNTs	15	6.0	53
hG@SnS bundles	4.5	3.82	54
B‐SnS_2_	3.98	3.7	55
Ni_3_V_2_O_8_/CC	4	2.6	56
CoP@PPy NWs/CP	1.9	1.3	57
SnS‐G	1.0	1.04	58
Sb@TiO_2−_ *_x_* nanotubes	1.5	1.0	59
TiO_2_@G nanocomposites	2.5	0.66	60
FeS/C	2.0	1.24	61
CaV_4_O_9_ microflowers	3.6	1.0	62
SnO_2_@SnS	1.2	0.87	63
N‐GF	1.0	1.1	64

Moreover, the volumetric capacity (*Q*_v_) of CNT/CoSe_2_/C hybrid sponge was also computed using the formula *Q*_v_ = *Q*_M_/*A*×*h*, where *Q*_M_, *A*, and *h* are the gravimetric capacity, base area, and thickness of the electrode, respectively. We obtained volumetric capacities of 61.3, 95.5, 112.0, 151.3, and 185.2 mAh cm^−3^ that correspond to bulk densities of 0.12, 0.20, 0.25, 0.35, and 0.46 mg cm^−3^, respectively, of CNT/CoSe_2_/C sponges (Figure S9a, Supporting Information). Figure S9b (Supporting Information) indicates that high volumetric capacity and density would be achieved by using initial larger sponge thickness. Additionally, we have also added some published results on the volumetric capacity comparison in particular and results are summarized in Table S3 (Supporting Information). It is obvious from the table that our volumetric capacity (185.2 mAh cm^−3^) is higher than most of the reported literature values and this is ascribed to higher tap density of hybrid sponge (Table S3, Supporting Information). However, some reported electrodes also showed higher volumetric capacity than our CNT/CoSe_2_/C electrode because of larger thickness of our electrode (0.34–0.38 mm) to maintain high stability by providing continuous pathway for mass flow.

The electrochemical kinetics insights of the electrodes after Na^+^ insertion were further investigated by CV at various scan rates and electrochemical impedance spectroscopy (EIS) analysis. **Figure** **7**a shows the CV curves of the CNT/CoSe_2_/C electrode after 100 cycles at various scan rates. All CV curves almost show identical behavior and consist of three reductions and one oxidation peak. The relationship between the peak current (*i*) and voltage (*v*) scan rates is given as

(3) $$\log\left(i\right)=b\times \log\left(v\right)+\log\left(a\right)$$

**Figure 7:**
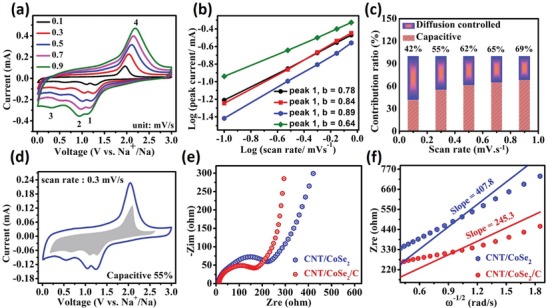
Quantitative capacitive storage analysis and diffusion kinetics of sodium ions in hybrid sponge electrodes: a) CV curves of CNT/CoSe_2_/C at various scan rates. b) The relationship between logarithm scan rates and logarithm peak current for all corresponding peaks. c) The contribution of capacitive capacities in percentage at different scan rates. d) Pseudocapacitive contribution (shaded region) in CV curve of CNT/CoSe_2_/C at 0.3 mV s^−1^. e) EIS spectra and f) graph of *Z*_re_ with ω^−1/2^ in the low frequency range of CNT/CoSe_2_ and CNT/CoSe_2_/C anodes after 30 cycles.

In Equation (3), the value of *b* is the most important parameter as explains the charge storage behavior (diffusion controlled if *b* ≤ 0.5 or pseudocapacitive if *b* ≥ 1) in the electrode material. The value of *b* was determined by the slope of Equation (3) [log(*v*) vs log (*i*)] and it is 0.78, 0.84, 0.89, and 0.64 for the peaks 1, 2, 3, and 4, respectively (Figure 7b). The value of *b* for all peaks is greater than 0.5 and near to 1 suggesting mixed behavior, i.e., diffusion controlled and pseudocapacitive. Such properties are useful for better electrochemical ion storage.^65^, ^66^ The exact pseudocapacitive and diffusion controlled contribution at a fixed scan rate can be determined by Equation (4)

(4) $$i=k_{1}\left(v\right)+k_{2}\left(v^{1/2}\right)$$

where *k*_1_ and *k*_2_ are equation parameters and their values can be determined by the slope and intercept of Figure S10 (Supporting Information) (*iv^−^* ^1/2^ vs *v* ^1/2^). The fraction of capacitive behavior is provided by the quantity *k*_1_(*v*) while diffusion controlled behavior is provided by the term *k*_2_(*v* ^1/2^). Figure 7c shows the trend of measured pseudocapacitive behavior that increases with increasing the scan rate. In addition, at a scan rate of 0.3 mV s^−1^ pseudocapacitive contribution is provided by the shaded region in Figure 7d.

Furthermore, the effect of carbon coating in the composites was also evaluated using EIS analysis after battery testing. Nyquist plots of the CNT/CoSe_2_ and CNT/CoSe_2_/C battery electrodes after 30 cycles as an anode of SIB are shown in Figure 7e, which delineate that R_ct_ of the CNT/CoSe_2_ electrode is higher than the CNT/CoSe_2_/C electrode (Table S3, Supporting Information). This indicates the continuous formation of SEI and the lack of electronic conductivity on the broken NPs surface during insertion/de‐insertion in case of CNT/CoSe_2_, resulting in poor cycling life. The circuit diagram to fit the EIS data is drawn and presented in Figure S11 (Supporting Information). Typically, *R*_s_, CPE‐1, and *R*_f_ show the ohmic resistance of the electrolyte, constant phase element (CPE), and SEI layer resistance, respectively, in the low frequency region. *R*_ct_ and CPE‐2 are attributed to charge transfer resistance and CPE of the electrolyte/electrode interface, respectively, whereas *Z*_w_ presents the sodium‐ion diffusion process in low frequency region. In this region, the Na^+^ diffusivity (*D*_Na_) of the electrodes can be calculated using Equation (5)

(5) $$D_{\text{Na}}=R^{2}T^{2}\text{/}2A^{2}n^{4}F^{4}C_{\text{Na}}{}^{2}\;\sigma_{\omega }{}^{2}$$

In Equation (5), *R*, *T*, *A*, *n*, *F*, *C*_Na_, and σ_ω_ are the universal gas constant, absolute temperature, the surface area of the electrode, number of electron transfer during oxidation, Faraday's constant, molar concentration of Na^+^ in the electrolyte (moles per cubic centimeter), and Warburg factor, respectively. All the terms used in Equation (5) are constant except σ_ω_ which can be obtained from the slope of *Z*_re_ versus ω^−1/2^ (Figure 7f), using Equation (6)

(6) $$Z_{\text{re}}=\;\text{Re}+R_{\text{ct}}+\sigma_{\omega }\omega^{-1\text{/}2}$$

Using the preceding calculation, the σ_ω_ and *D*_Na_ values of two composites are listed in Table S3 (Supporting Information). For CNT/CoSe_2_/C electrode, the *D*_Na_ value (2.3 × 10^−10^ cm^2^ s^−1^) is ≈2.76 times higher than that of CNT/CoSe_2_ (8.33 × 10^−11^ cm^2^ s^−1^), respectively. The EIS result in the high and low frequency region of the Nyquist plot shows that both the electronic conductivity and ionic diffusivity would significantly affect the electrochemical performance of CNT/CoSe_2_/C‐based electrodes. Without carbon overcoat on the NPs, the electronic conductivity and ionic transport are lower, consequently lower the electrochemical performance.

We further investigated the structural analysis of CNT/CoSe_2_/C electrodes after battery testing. CoSe_2_ NPs are still tightly deposited on CNT and the original morphology of the hybrid is maintained (Figure S12a,b, Supporting Information). From Figure S12c (Supporting Information), C‐overcoat and CoSe_2_ NPs with the size of ≈25–30 nm coated on CNT can be clearly seen without agglomeration of NPs. HRTEM reveals that CoSe_2_ have transformed into amorphous after long battery cycling (Figure S12, Supporting Information). The repeated insertion–extraction of Na^+^ in the active materials may turn the highly crystalline structure into amorphous that improved the electrochemical process.^43^

In summary, a dual protection strategy is applied on CNT/CoSe_2_/C electrode to achieve high cyclic stability, mass loading, and areal capacity. The Co_3_O_4_ NPs grafted onto CNT are converted into CoSe_2_ using safe and environmental friendly hydrothermal/calcination process with core–shell structure (CNT/CoSe_2_) and then intertwined with ≈4 nm thick carbon overcoat by carbonization approach. The CNT/CoSe_2_/C presented a high discharge capacity of 531 mAh g^−1^ at 100 mA g^−1^ after 100 cycles. Moreover, it was densified by compaction with preserving its 3D networks and morphology due to high inherent porosity of hybrid sponge. Using this approach a high areal capacity up to 7.04 mAh cm^−2^ corresponds to an ultrahigh areal loading (17.4 mg cm^−2^). The novel synthesis and improved areal capacity of the CNT/CoSe_2_/C sponge make it an appropriate anode material for practical energy storage devices.

## Experimental Section

Information about the synthesis of CNT/Co_3_O_4_, used characterization tools, and electrochemical methods are provided in the Supporting Information.

*Synthesis of CNT/CoSe_2_*: The CNT/Co_3_O_4_ was converted into CNT/CoSe_2_ by novel solvothermal/calcination process. In a typical synthesis, 4.0 mmole NaBH_4_ was first dissolved into 10 mL deionized (DI) water and then 2.0 mmole Se powder was mixed by magnetic stirring. Afterward, CNT/Co_3_O_4_ sponge was immersed into the freshly prepared solution and then the solution containing CNT/Co_3_O_4_ sponge transferred into a 20 mL Teflon lined autoclave for the solvothermal reaction at 180 °C for 10 h. After the reaction, the sponge was again washed with ethanol and DI water and then freeze‐dried. Finally, to remove the Se impurity and attain a highly crystalline structure of CoSe_2_, the hybrid sponge was annealed at 400 °C for 2 h and obtained highly crystalline CNT/CoSe_2_ sponge.

*Synthesis of CNT/CoSe_2_/C*: The CNT/CoSe_2_/C was obtained by the carbonization process. First, the as‐synthesized CNT/CoSe_2_ sponge was dipped into 1.0 m aqueous glucose solution and executed for hydrothermal reaction in an electric oven at 180 °C for 10 h. The glucose‐coated CNT/CoSe_2_ sponge was washed with ethanol and DI water to remove impurity. Finally, glucose‐coated CNT/CoSe_2_ sponge was carbonized by heat treatment at 450 °C for 2 h in the inert environment and the final product CNT/CoSe_2_/C was obtained.

## Conflict of Interest

The authors declare no conflict of interest.

## Supporting information

Supporting Information

## References

[1] E. C. Evarts , Nature 2015, 526, S93. 26509953 10.1038/526S93a

[2] W. Luo , F. Shen , C. Bommier , H. Zhu , X. Ji , L. Hu , Acc. Chem. Res. 2016, 49, 231. 26783764 10.1021/acs.accounts.5b00482

[3] H. Tabassum , R. Zou , A. Mahmood , Z. Liang , Q. Wang , H. Zhang , S. Gao , C. Qu , W. Guo , S. Guo , Adv. Mater. 2018, 30, 1705441. 10.1002/adma.201705441 29318669

[4] M. Yousaf , Y. Wang , Y. Chen , Z. Wang , A. Firdous , Z. Ali , N. Mahmood , R. Zou , S. Guo , R. P. S. Han , Adv. Energy Mater. 2019, 9, 1900567.

[5] H. Zhu , F. Zhang , J. Li , Y. Tang , Small 2018, 14, 1703951. 10.1002/smll.201703951 29399964

[6] M. Wang , Y. Tang , Adv. Energy Mater. 2018, 8, 1703320.

[7] M. A. Reddy , M. Helen , A. Groß , M. Fichtner , H. Euchner , ACS Energy Lett. 2018, 3, 2851.

[8] A. Mahmood , S. Li , Z. Ali , H. Tabassum , B. Zhu , Z. Liang , W. Meng , W. Aftab , W. Guo , H. Zhang , M. Yousaf , S. Gao , R. Zou , Y. Zhao , Adv. Mater. 2019, 31, 1805430. 10.1002/adma.201805430 30422332

[9] K. Zhang , M. Park , L. Zhou , G.‐H. Lee , W. Li , Y.‐M. Kang , J. Chen , Adv. Funct. Mater. 2016, 26, 6728.

[10] J. Li , D. Yan , T. Lu , Y. Yao , L. Pan , Chem. Eng. J. 2017, 325, 14.

[11] X. Ma , L. Zou , W. Zhao , Chem. Commun. 2018, 54, 10507. 10.1039/c8cc04426k 30167610

[12] Y. Fang , X. Y. Yu , X. W. D. Lou , Adv. Mater. 2018, 30, 1706668. 10.1002/adma.201706668 29633418

[13] N. Liu , Z. Lu , J. Zhao , M. T. McDowell , H. W. Lee , W. Zhao , Y. Cui , Nat. Nanotechnol. 2014, 9, 187. 24531496 10.1038/nnano.2014.6

[14] J. Liang , X. Y. Yu , H. Zhou , H. B. Wu , S. Ding , X. W. Lou , Angew. Chem., Int. Ed. 2014, 53, 12803. 10.1002/anie.201407917 25251871

[15] M. Singh , J. Kaiser , H. Hahn , J. Electrochem. Soc. 2015, 162, A1196.

[16] J. Liang , H. Hu , H. Park , C. Xiao , S. Ding , U. Paik , X. W. Lou , Energy Environ. Sci. 2015, 8, 1707.

[17] Y. Gogotsi , P. Simon , Science 2011, 334, 917. 22096182 10.1126/science.1213003

[18] K. G. Gallagher , S. E. Trask , C. Bauer , T. Woehrle , S. F. Lux , M. Tschech , P. Lamp , B. J. Polzin , S. Ha , B. Long , Q. Wu , W. Lu , D. W. Dees , A. N. Jansen , J. Electrochem. Soc. 2016, 163, A138.

[19] Y. Sun , N. Liu , Y. Cui , Nat. Energy 2016, 3, 16071.

[20] M. Yousaf , H. T. H. Shi , Y. Wang , Y. Chen , Z. Ma , A. Cao , H. E. Naguib , R. P. S. Han , Adv. Energy Mater. 2016, 6, 1600490.

[21] S.‐H. Park , P. J. King , R. Tian , C. S. Boland , J. Coelho , C. Zhang , P. McBean , N. McEvoy , M. P. Kremer , D. Daly , J. N. Coleman , V. Nicolosi , Nat. Energy 2019, 4, 560.

[22] H. Sun , J. Zhu , D. Baumann , L. Peng , Y. Xu , I. Shakir , Y. Huang , X. Duan , Nat. Rev. Mater. 2019, 4, 45.

[23] X. Gui , J. Wei , K. Wang , A. Cao , H. Zhu , Y. Jia , Q. Shu , D. Wu , Adv. Mater. 2010, 22, 617. 20217760 10.1002/adma.200902986

[24] Y. Wang , Z. Ma , Y. Chen , M. Zou , M. Yousaf , Y. Yang , L. Yang , A. Cao , R. P. Han , Adv. Mater. 2016, 28, 10175. 27690278 10.1002/adma.201603812

[25] Z. Ma , Y. Wang , Y. Yang , M. Yousaf , M. Zou , A. Cao , R. P. S. Han , RSC Adv. 2016, 6, 30098.

[26] Y. Chen , Y. Wang , M. Yousaf , Z. Ma , M. Zou , A. Cao , R. P. S. Han , Mater. Res. Bull. 2017, 93, 1.

[27] Z. Wang , Y. Wang , Y. Chen , M. Yousaf , H. Wu , A. Cao , R. P. S. Han , Adv. Funct. Mater. 2019, 29, 1807467.

[28] H. Azimi , Y. Hou , C. J. Brabec , Energy Environ. Sci. 2014, 7, 1829.

[29] H. Shi , X. Zhou , Y. Lin , X. Fu , Mater. Lett. 2008, 62, 3649.

[30] A. R. M. d. Oliveira , L. Piovan , F. Simonelli , A. Barison , M. F. C. Santos , M. B. M. de Mello , J. Organomet. Chem. 2016, 806, 54.

[31] B. Yu , F. Qi , Y. Chen , X. Wang , B. Zheng , W. Zhang , Y. Li , L. C. Zhang , ACS Appl. Mater. Interfaces 2017, 9, 30703. 28829113 10.1021/acsami.7b09108

[32] S. K. Park , Y. C. Kang , ACS Appl. Mater. Interfaces 2018, 10, 17203. 29717862 10.1021/acsami.8b03607

[33] S.‐K. Park , J. K. Kim , Y. C. Kang , Chem. Eng. J. 2017, 328, 546.

[34] J. Yang , G.‐H. Cheng , J.‐H. Zeng , S.‐H. Yu , X.‐M. Liu , Y.‐T. Qian , Chem. Mater. 2001, 13, 848.

[35] D. Kong , H. Wang , Z. Lu , Y. Cui , J. Am. Chem. Soc. 2014, 136, 4897. 24628572 10.1021/ja501497n

[36] Y. Liu , H. Cheng , M. Lyu , S. Fan , Q. Liu , W. Zhang , Y. Zhi , C. Wang , C. Xiao , S. Wei , B. Ye , Y. Xie , J. Am. Chem. Soc. 2014, 136, 15670. 25310506 10.1021/ja5085157

[37] J. Yang , H. Gao , S. Men , Z. Shi , Z. Lin , X. Kang , S. Chen , Adv. Sci. 2018, 5, 1800763. 10.1002/advs.201800763 PMC6299709 30581698

[38] H. Tabassum , C. Zhi , T. Hussain , T. Qiu , W. Aftab , R. Zou , Adv. Energy Mater. 2019, 9, 1901778.

[39] Q. Yu , B. Jiang , J. Hu , C. Y. Lao , Y. Gao , P. Li , Z. Liu , G. Suo , D. He , W. A. Wang , G. Yin , Adv. Sci. 2018, 5, 1800782. 10.1002/advs.201800782 PMC6193164 30356990

[40] G. Ma , C. Li , F. Liu , M. K. Majeed , Z. Feng , Y. Cui , J. Yang , Y. Qian , Mater. Today Energy 2018, 10, 241.

[41] H. Wang , X. Wang , D. Yang , B. Zheng , Y. Chen , J. Power Sources 2018, 400, 232.

[42] C. Cui , Z. Wei , G. Zhou , W. Wei , J. Ma , L. Chen , C. Li , J. Mater. Chem. A 2018, 6, 7088.

[43] M. Yousaf , Y. Wang , Y. Chen , Z. Wang , W. Aftab , A. Mahmood , W. Wang , S. Guo , R. P. S. Han , ACS Appl. Mater. Interfaces 2018, 10, 14622. 29652482 10.1021/acsami.7b19739

[44] C. An , Y. Yuan , B. Zhang , L. Tang , B. Xiao , Z. He , J. Zheng , J. Lu , Adv. Energy Mater. 2019, 9, 1900356.

[45] Z. Li , H. Xue , J. Wang , Y. Tang , C.‐S. Lee , S. Yang , ChemElectroChem 2015, 2, 1682.

[46] J. S. Cho , J.‐S. Park , K. M. Jeon , Y. C. Kang , J. Mater. Chem. A 2017, 5, 10632.

[47] Y. N. Ko , S. H. Choi , Y. C. Kang , ACS Appl. Mater. Interfaces 2016, 8, 6449. 26918934 10.1021/acsami.5b11963

[48] G. D. Park , Y. C. Kang , Chem. ‐ Eur. J. 2016, 22, 4140.26864320

[49] H. Hou , X. Qiu , W. Wei , Y. Zhang , X. Ji , Adv. Energy Mater. 2017, 7, 1602898.

[50] V. Sharova , A. Moretti , G. Giffin , D. Carvalho , S. Passerini , C 2017, 3, 22.

[51] Y. Chen , Y. Wang , Z. Wang , M. Zou , H. Zhang , W. Zhao , M. Yousaf , L. Yang , A. Cao , R. P. S. Han , Adv. Energy Mater. 2018, 8, 1702981.

[52] H. Ye , L. Wang , S. Deng , X. Zeng , K. Nie , P. N. Duchesne , B. Wang , S. Liu , J. Zhou , F. Zhao , N. Han , P. Zhang , J. Zhong , X. Sun , Y. Li , Y. Li , J. Lu , Adv. Energy Mater. 2017, 7, 1601602.

[53] Y. Liu , X. He , D. Hanlon , A. Harvey , J. N. Coleman , Y. Li , ACS Nano 2016, 10, 8821. 27541502 10.1021/acsnano.6b04577

[54] D. Chao , B. Ouyang , P. Liang , T. T. T. Huong , G. Jia , H. Huang , X. Xia , R. S. Rawat , H. J. Fan , Adv. Mater. 2018, 30, 1804833. 10.1002/adma.201804833 30302835

[55] D. Chao , P. Liang , Z. Chen , L. Bai , H. Shen , X. Liu , X. Xia , Y. Zhao , S. V. Savilov , J. Lin , Z. X. Shen , ACS Nano 2016, 10, 10211. 27768284 10.1021/acsnano.6b05566

[56] C. Zhou , S. Fan , M. Hu , J. Lu , J. Li , Z.‐H. Huang , F. Kang , R. Lv , J. Mater. Chem. A 2017, 5, 15517.

[57] J. Zhang , K. Zhang , J. Yang , G.‐H. Lee , J. Shin , V. W.‐h. Lau , Y.‐M. Kang , Adv. Energy Mater. 2018, 8, 1800283.

[58] T. Zhou , W. K. Pang , C. Zhang , J. Yang , Z. Chen , H. K. Liu , Z. Guo , ACS Nano 2014, 8, 8323. 25010575 10.1021/nn503582c

[59] N. Wang , Z. Bai , Y. Qian , J. Yang , Adv. Mater. 2016, 28, 4126. 26923105 10.1002/adma.201505918

[60] C. Chen , Y. Wen , X. Hu , X. Ji , M. Yan , L. Mai , P. Hu , B. Shan , Y. Huang , Nat. Commun. 2015, 6, 6929. 25906991 10.1038/ncomms7929

[61] Y. X. Wang , J. Yang , S. L. Chou , H. K. Liu , W. X. Zhang , D. Zhao , S. X. Dou , Nat. Commun. 2015, 6, 8689. 26507613 10.1038/ncomms9689 PMC4846313

[62] X. Xu , P. Wu , Q. Li , W. Yang , X. Zhang , X. Wang , J. Meng , C. Niu , L. Mai , Nano Energy 2018, 50, 606.

[63] Y. Zheng , T. Zhou , C. Zhang , J. Mao , H. Liu , Z. Guo , Angew. Chem., Int. Ed. 2016, 55, 3408. 10.1002/anie.201510978 26844806

[64] J. Xu , M. Wang , N. P. Wickramaratne , M. Jaroniec , S. Dou , L. Dai , Adv. Mater. 2015, 27, 2042. 25689053 10.1002/adma.201405370

[65] F. Niu , J. Yang , N. Wang , D. Zhang , W. Fan , J. Yang , Y. Qian , Adv. Funct. Mater. 2017, 27, 1700522.

[66] Z. Ali , T. Tang , X. Huang , Y. Wang , M. Asif , Y. Hou , Energy Storage Mater. 2018, 13, 19.

