# Supplementary material for: A Dual Protection System for Heterostructured 3D CNT/CoSe2/C as High Areal Capacity Anode for Sodium Storage
Source: Adv Sci (Weinh). 2020 Jan 21;7(5):1902907. doi: 10.1002/advs.201902907 (PMC7055556; doi:10.1002/advs.201902907)
Supplement: Supplementary file 1 — Supporting Information [file ADVS-7-1902907-s001.pdf]

Copyright WILEY-VCH Verlag GmbH & Co. KGaA, 69469 Weinheim, Germany, 2020.

ADVANCED SCIENCE
Open Access

Supporting Information

for *Adv. Sci.,* DOI: 10.1002/advs.201902907

A Dual Protection System for Heterostructured 3D CNT/ $CoSe_2$/C as High Areal Capacity Anode for Sodium Storage

Muhammad Yousaf, Yijun Chen, Hassina Tabassum, Zhipeng Wang, Yunsong Wang, Adeel Y. Abid, Asif Mahmood, Nasir Mahmood, Shaojun Guo, Ray P. S. Han, and Peng Gao**

Supporting Information

A Dual Protection System for Heterostructured 3D CNT/$CoSe_2$/C as High Areal Capacity Anode for Sodium Storage

Muhammad Yousaf, Yijun Chen, Hassina Tabassum, Zhipeng Wang, Yunsong Wang, Adeel Y Abid, Asif Mahmood, Nasir Mahmood, Shaojun Guo, Ray P.S. Han*, Peng Gao**

Dr. M. Yousaf, Y. Chen, Dr. H. Tabassum, Z. Wang, Y. Wang, Prof. S. Guo, Prof. R.P.S. Han
*E-mail: guosj@pku.edu.cn (S.G), ray-han@pku.edu.cn (R.P.S.H.)
Department of Material Science and Engineering, Peking University, Beijing 100871, China
Dr. M. Yousaf, A.Y. Abid, Prof. P. Gao
International Center for Quantum Materials and Electron Microscopy Laboratory, School of Physics, Peking University, Beijing 100871, China
*E-mail: p-gao@pku.edu.cn (P.G),
Dr. A. Mahmood
School of Chemical and Biomolecular Engineering, The University of Sydney, 2006, Sydney, Australia
Dr. N. Mahmood
School of Engineering, RMIT University, 124 La Trobe Street, Melbourne, Victoria, 3001, Australia
Keywords: sodium ion battery, dual conductive network, 3D electrodes, CNT/$CoSe_2$/C, high areal capacity

Table of Content

Detailed experimental methods
Procedure to calculate the content of carbon in various hybrids
The electrochemical response of pure CNT sponge
Figure S1
Figure S2
Figure S3
Figure S4
Figure S5
Figure S6
Figure S7
Figure S8
Figure S9
Figure S10
Figure S11
Figure S12
Table S1
Table S2
Table S3
Table S4

Detailed Experimental Methods

All the chemicals were of analytical grade and used without further purification. The fabrication process of the CNT/$CoSe_2$/C sponge consists of three major steps as described below.

***Synthesis of CNT/Co_3O_4*:** The CNT sponge was first fabricated by an already reported method by Gui et al.[1] using ferrocene and 1,2-dichlorobenzene as catalyst and carbon source. To attach some oxygen functional group on CNTs, the as-prepared CNT sponge was first treated with concentrated nitric acid (HNO_3) at 120 ^oC for 10 hours and washed with DI water several time. The Co_3O_4 NPs was then decorated on CNTs by the simple hydrothermal process as presented in our previous work.[2] Typically, 1.0 mol L^{-1} concentrated aqueous solution containing $Co(No_3)_2.6H_2O$ was prepared by magnetic stirring followed by adding an excess amount of concentrated ammonia (HNo_3) until the solution turned to transparent. Next, a piece of CNT sponge (100 mg) was dipped in the already prepared solution for ~20 minutes and then taken out. Afterward, the CNT sponge containing Co precursor was placed in Teflon stainless autoclave and executed for hydrothermal reaction in an electric oven at 120 ^oC for 12 hours. After completing the reaction the sponge was washed with ethanol and DI water several times and finally, it was freeze-dried and got CNT/Co_3O_4.

***Structural Characterizations*:** X-ray diffractometer (Burker D8, XRD) with Cu_α radiations was used to obtain the crystal structure of all the products. Raman spectra were achieved using Horiba (HR800) in Via Raman microscope using 514 nm laser light. The oxidation states and surface chemical compositions in the products were obtained by X-ray photoelectron microscopy (XPS) using AXIS Supra (Shimadzu Group, UK Kratos) with monochromatized Al K_α radiation. The structure and surface morphology of the products were examined by scanning electron microscopy (FEI Nova Nano SEM 430) and transmission electron microscopy (FEI Tecnai T20) equipped with an EDX spectrometer. Thermogravimetric analysis (TGA) was used to determine the content of carbon in each product with a temperature range from 25 ^oC to 700 ^oC in the air. The Brunauer-Emmett-Teller (BET) test (Tristar 3020 USA) was used to calculate the surface area at 77 K, while the nonlinear density functional theory (NL-DFT) method was used to determine the pore size distribution.

***Electrochemical measurements*:** The coin-type cells (CR 2032) were manufactured in a glove box containing Ar gas with O_2 contents > 1.0 ppm and H_2O > 0.5. The as-fabricated sponges were cut into required shapes and heights and were used as free standing anodes without using dead elements (like a current collector, binders and conductive carbon). The Na metal was cut into circular discs and was used as a reference electrode (cathode). The glass fiber filter paper (GF/D, Whatman) was served as a separator, whereas 1.0 M $NaClO_4$ with 5% ethylene carbonate (EC) was employed as the electrolyte. The cyclic voltammetry (CV) curves at various scan rates and electrochemical impedance spectroscopy (EIS) graphs were determined using ChI660E electrochemical work station. The discharge/charge voltage profiles, cyclic tests, and rate performance graphs were achieved by LAND CT 2001A analyzer. For ex-situ tests such as TEM, HRTEM of anodes, the cells were de-assembled in Ar glove box and then anodes were washed with DMC and ethanol and finally dried in a vacuum oven at 70 ^oC. For the calculation of gravimetric capacity active mass ($CoSe_2$) of the electrode was used which was ~3.4-4.0 mg/cm^2.

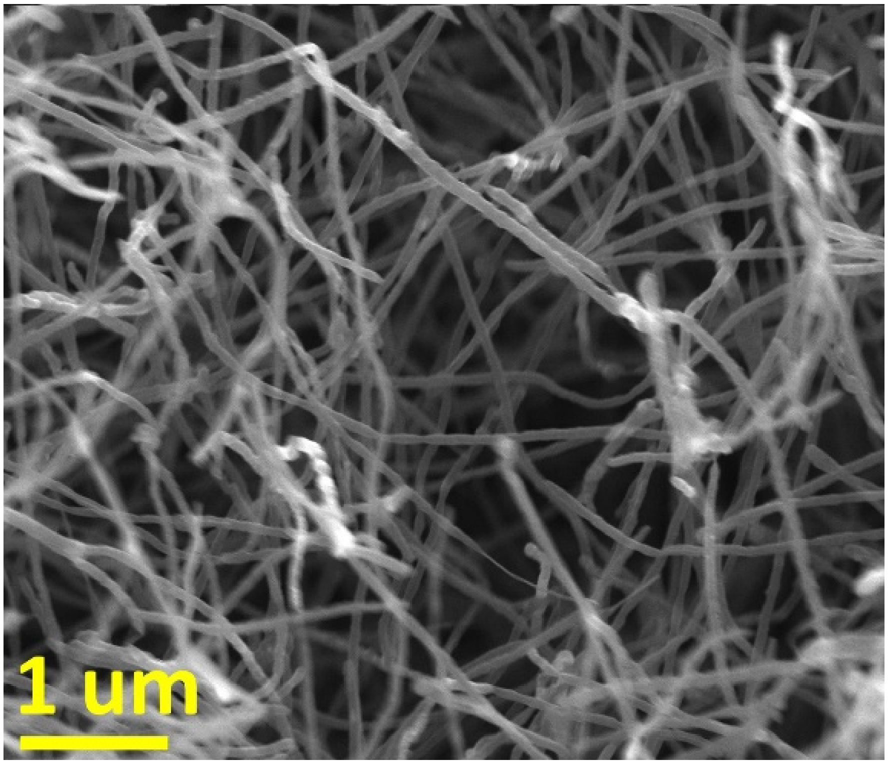

Figure S1. SEM micrograph of pure CNT sponge.

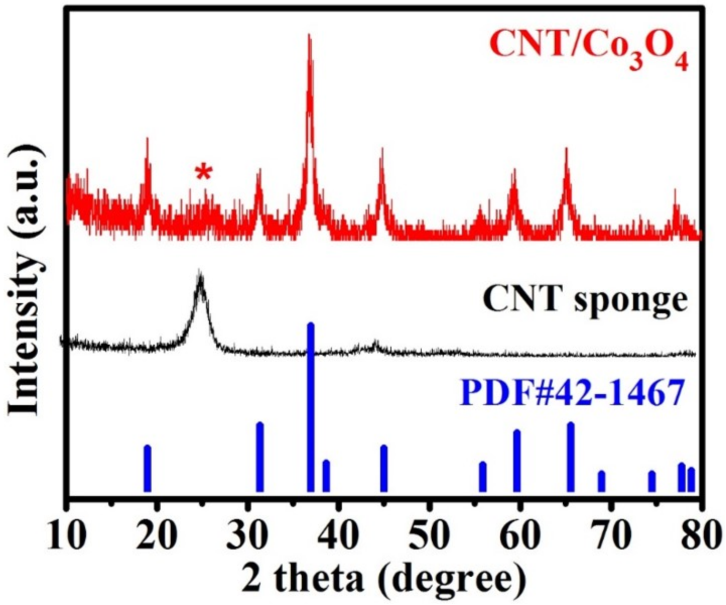

Figure S2. XRD pattern of pure CNT sponge and CNT/Co_3O_4

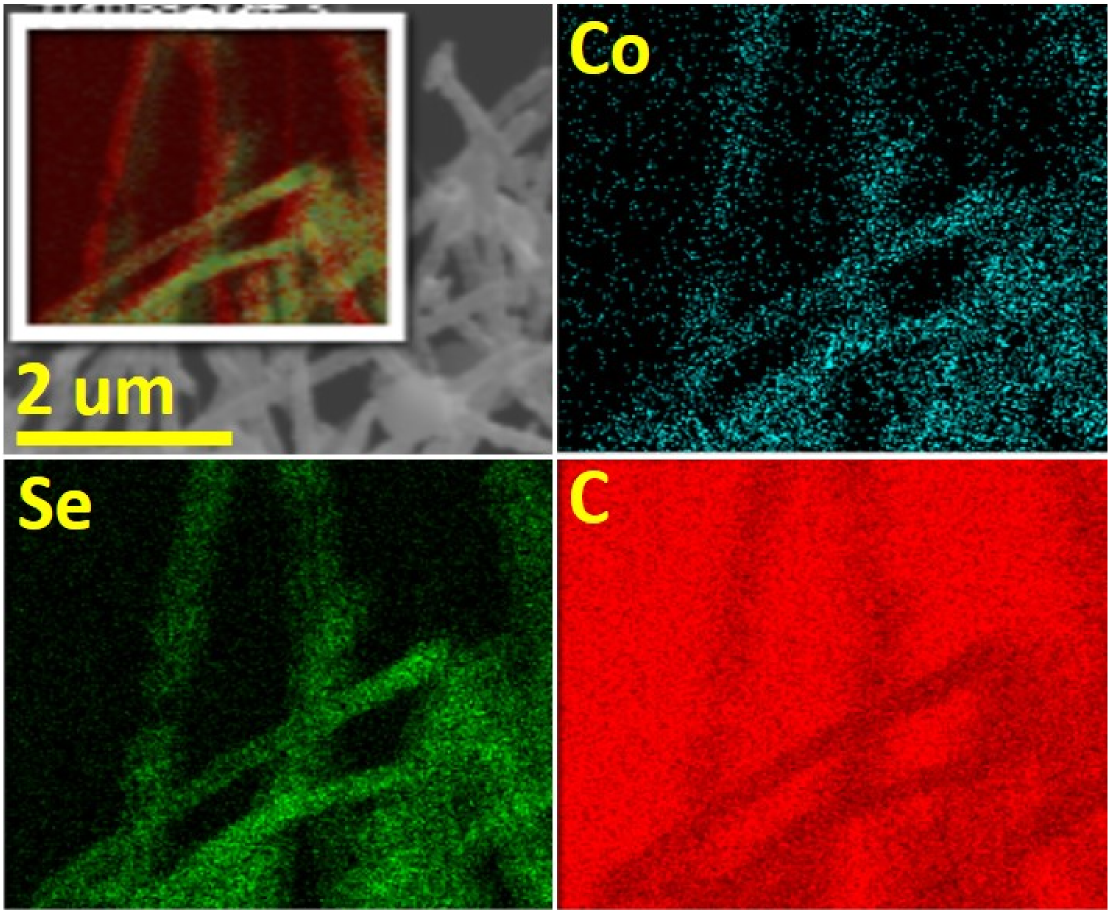

Figure S3. SEM-EDX mapping of CNT/$CoSe_2$/C.

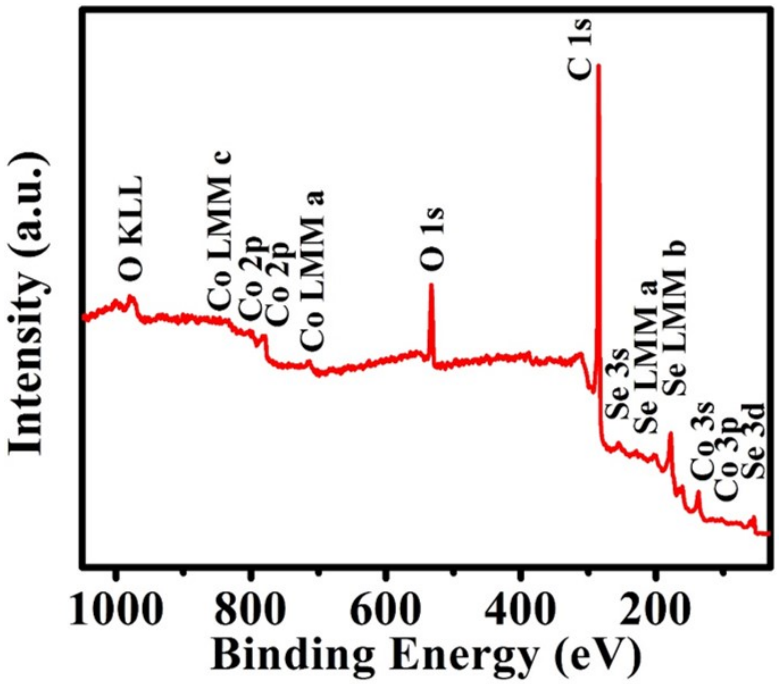

Figure S4. Survey spectrum of CNT/$CoSe_2$/C

Procedure to calculate the content of carbon in various hybrids:

Thermogravimetric analysis (TGA) was used to calculate the content of carbon in hybrids and is provided in **Figure 4e** in the manuscript that shows that the residual catalysts (Fe_2O_3) in pure CNT sponge is 3%. Whereas, in TGA curves for CNT/Co_3O_4, CNT/$CoSe_2$ and CNT/$CoSe_2$/C the residues is 65%, 31% and 28%, respectively (**Figure 4e** in the manuscript). The products in residues for hybrids mainly consisted of Co_3O_4 and some traces of Fe_2O_3. The content of carbon in various hybrids is calculated based on the following procedure[3]:

Molecular weight of Fe_2O_3 = M_{Fe2O3} = 159.86

Molecular weight of $CoSe_2$ = M_{CoSe2} = 216.85

Molecular weight of Co_3O_4 = M_{Co3O4} = 241

Amount of Fe in the composite = A

Amount of $CoSe_2$ in the composite = B

Amount of CNT in the composite = C

Pure CNT sponge contains residual catalyst of 3%, therefore 3% * 7/10 = A/(A+C)

Solve for A

A = C/46.619 (1)

For CNT/$CoSe_2$ Sponge:

The weight loss in CNT/$CoSe_2$ hybrid is about 69% (**Figure 4e** in the manuscript).

$69\% = B*(M_{CoSe2} – 1/3*M_{Co3O4}/M_{CoSe2} + C$

0.69 = B*(216.85-241/3)/216.85 + C

0.69 = 0.63*B + C (2)

The residue in the CNT/$CoSe_2$ hybrid is about 31% (**Figure 4e** in the manuscript).

$31\% = B*(M_{Co3O4})/(3*M_{CoSe2})) + M_{Fe2O3}$

$0.31 = B*80.33/216.85 + M_{Fe2O3}$

$0.31 = 0.37*B + M_{Fe2O3}$ (3)

Since,

$A/M_{Fe2O3} = 2*56/160$

Solve for A

$A = 0.7*(M_{Fe2O3})$ (i)

$3\% = M_{Fe2O3}/(A + C)$

$0.3 = M_{Fe2O3}/(A + C)$ (ii)

Put the value of A in Equation (ii)

$0.3= M_{Fe2O3}/(0.7* M_{Fe2O3} + C)$

Solve for M_{Fe2O3}

$M_{Fe2O3} = C/32.63$ (iii)

Insert Equation (iii) into Equation (3)

10.1153 = 12.0731*Y + Z (4)

Subtract Equation (4) from Equation (2)

9.4253 = 11.4431*B and solve for B = 0.8237

Insert the value of B in Equation (2)

0.69 = (0.63)(0.8237) + C and solve for C = 0.1107

Put the value of C in Equation (1) to get A = 0.1107/46.619 = 0.00024

Amount of carbon in the CNT/$CoSe_2$ hybrid = [0.1107/(A+ B + C)]*100

Amount of carbon in the CNT/$CoSe_2$ hybrid = 0.1107/(0.93464)*100

Amount of carbon in the CNT/$CoSe_2$ hybrid = 11.84%

Similarly, according to the above procedure, the amount of carbon in CNT/Co_3O_4 and CNT/$CoSe_2$/C is 35.07% and 25.6%, respectively.

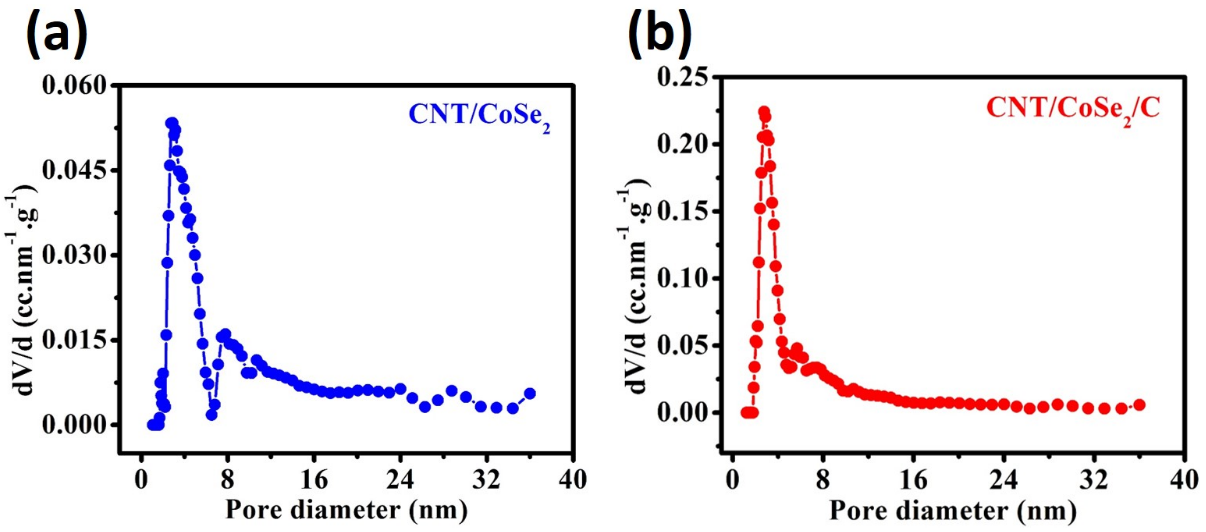

Figure S5. Pore size distributions of (a) CNT/$CoSe_2$ (b) CNT/$CoSe_2$/C, respectively

Table S1. N_2 adsorption/desorption results of different materials.

**Materials**	**Surface area (m^2/g)**	**Pore diameter (nm)**	**Pore volume (cc/g)**
CNT/$CoSe_2$	50.24	2.89	0.162
CNT/$CoSe_2$/C	74.01	2.77	0.171

Table S2. Comparison of charge capacities of CNT/$CoSe_2$/C and CNT/$CoSe_2$ anodes

**Electrode materials**	**1st charge capacity (mAh/g)**	**2nd charge capacity (mAh/g)**	**3rd charge capacity (mAh/g)**	**4th charge capacity (mAh/g)**
CNT/$CoSe_2$	535	496	474	457
CNT/$CoSe_2$/C	544	439	537	536

The electrochemical response of pure CNT sponge as an anode for SIB:

The electrochemical response of pure CNT sponge as a free-standing anode for SIB is presented in **Figure S6**. First, cyclic voltammetry (CV) test was carried out to study the electrochemical response of pure CNT sponge. **Figure S6a** shows the CV curves for the first three cycles under a scan rate of 0.3 mV/s in the potential range of 0.01-3.0 V. Three reduction peaks located at 0.51, 0.31 and 0.01 V can be observed in the first cathodic process. The strongest reduction peak at 0.51 V in the subsequent second and third cycles vanishes due to the decomposition of the electrolyte and irreversible formation of solid electrolyte interface (SEI) film on the surface of the electrode.[4-5] The reduction peak at 0.01 V is attributed to the intercalation and adsorption of Na^+ ions into the carbon.[6] Meanwhile one anodic peak at 0.46 V occurs during all three oxidation cycles. Galvanostatic charge/discharge voltage profile was carried out in the voltage window of 0.01-3.0 V at the current density of 100 mAh/g to further investigate the electrochemical behavior of pure CNT sponge (**S6b**). In the first cycle, the CNT sponge displays the discharge and charge capacities of 579 and 93 mAh/g, respectively. The large irreversible capacity in initial cycles can be ascribed to the electrolyte decomposition and formation of SEI layer on the surface of electrode at low potential and it is unavoidable phenomenon in porous carbon based materials.[7] Moreover, it can be observed that the charge/discharge voltage profiles of the 2nd, 3rd and 4th cycles overlap each other (**S6b**), showing the good reversibility of Na^+ ions insertion/extraction in pure CNT sponge electrode as well. The long cyclic stability of pure CNT sponge was carried out at current density of 500 mA/g for 500 cycles as shown in **Figure S6c**. After 500 cycles, the stable discharge capacity of 75.5 mAh/g was achieved that indicates the high stability of CNT sponge. The Coulombic efficiency of CNT sponge is provided in **Figure S6c** with a Coulombic efficiency of 16.11 % for the first cycle. The low Coulombic efficiency in the first cycle is attributed to high porosity and surface area of pure CNT sponge.[1, 6] After the first cycle, the Coulombic efficiency is improved and close to 100 % in subsequent cycles, which confirms the formation of a stable SEI layer (**Figure S6c**). To further explore the kinetics of Na^+ ions, electrochemical impedance (EIS) was carried out (**Figure S6d**). Before cycling the CNT sponge electrode showed a R_{ct} of 152 Ω that was decreased to 97 Ω after 50 cycles due to the formation of SEI film that gives the rapid routes for better transfer of electron and ion.

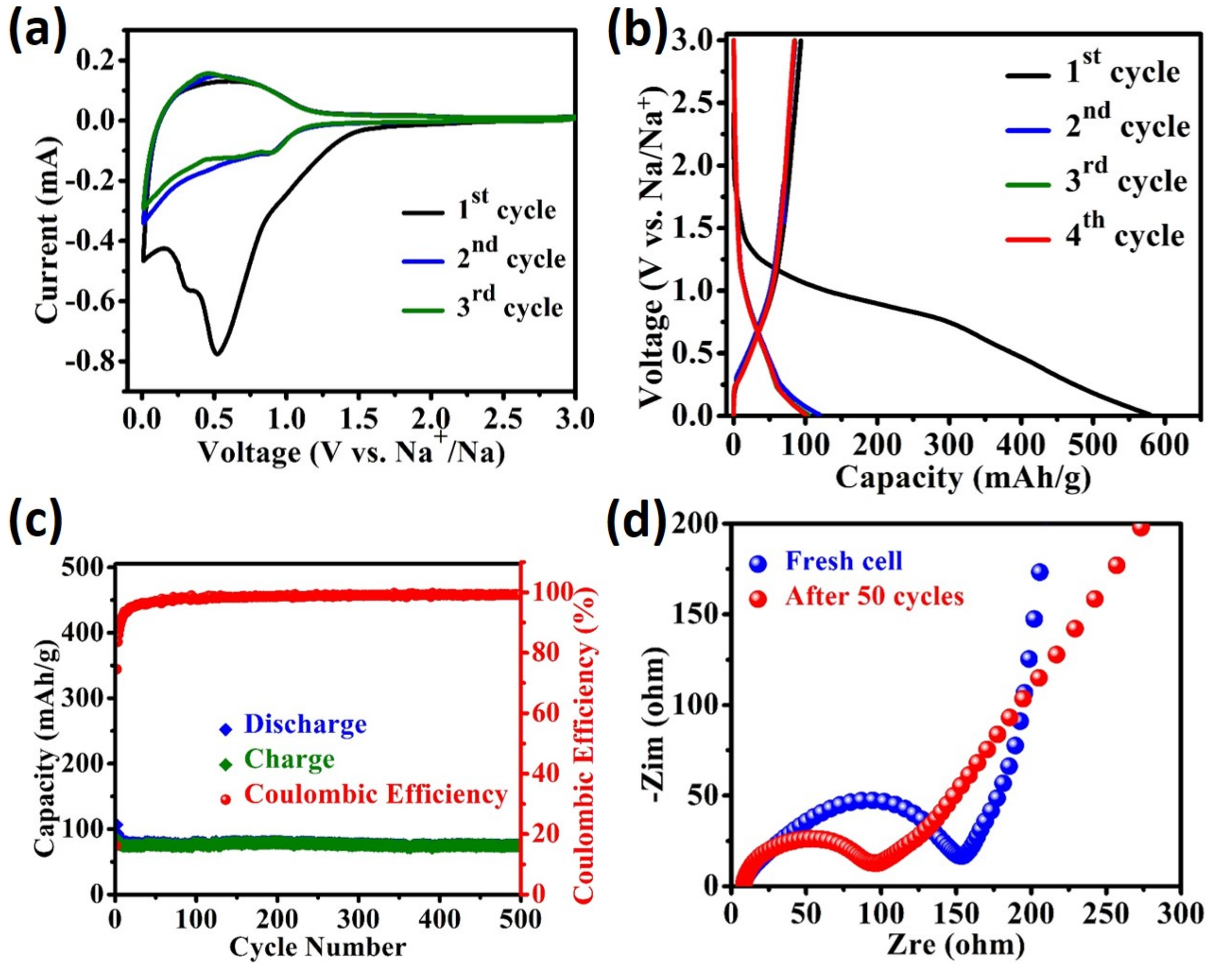

Figure S6. Electrochemical performance of CNT sponge: (a) CV curves at a scan rate of 0.3 mV/s (b) Discharge/charge voltage profile at 100 mA/g current density of 100 mA/g in voltage window 0.01-3.0 V. (c) Cyclic stability and Coulombic efficiency (d) EIS spectra.

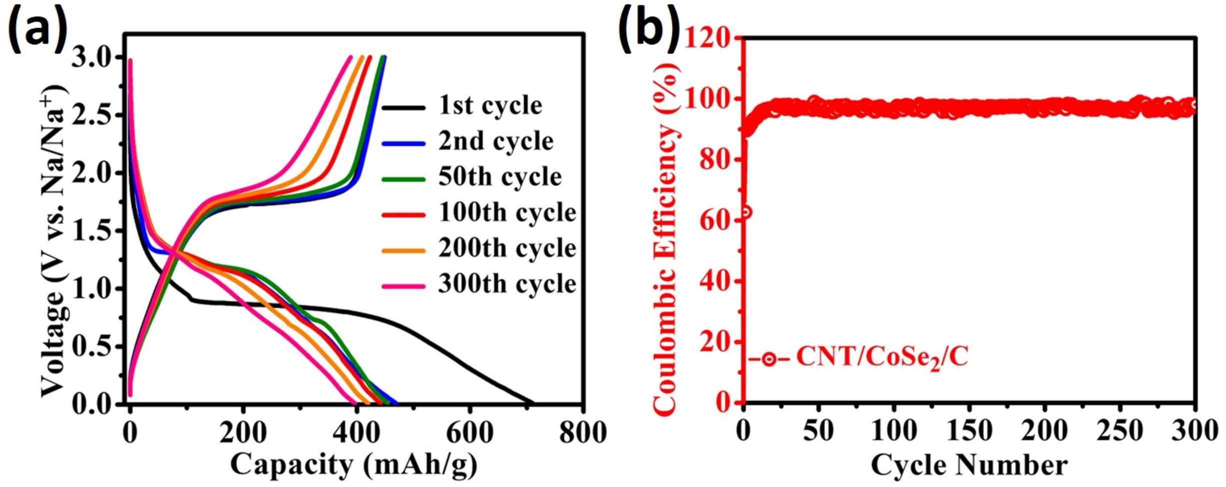

Figure S7. (a) Discharge/charge voltage profiles of CNT/$CoSe_2$/C electrode at 500 mA/g of various cycles, (b) Coulombic efficiency of CNT/$CoSe_2$/C anode.

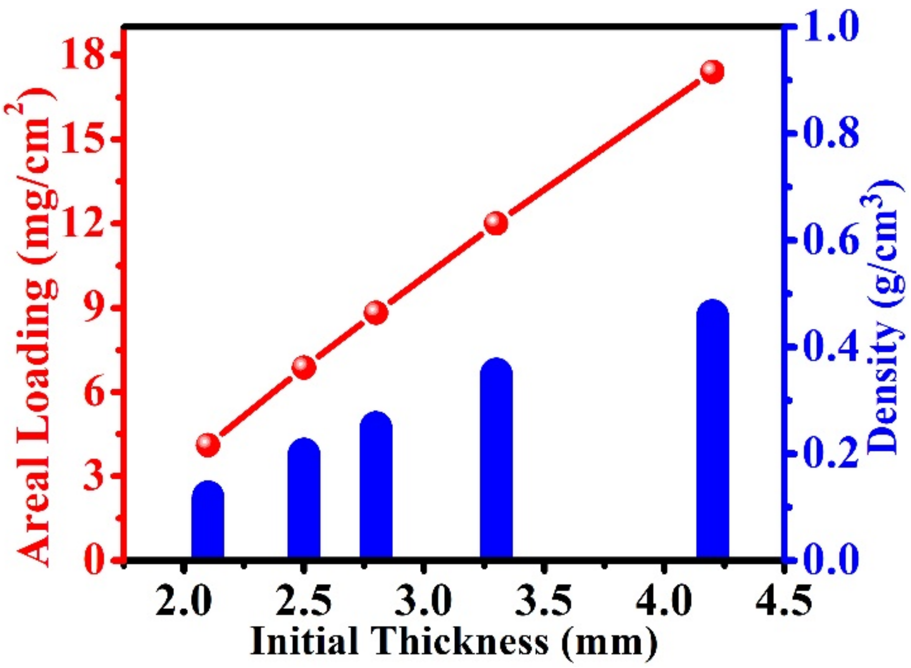

Figure S8. The relationship among initial thickness, areal mass loading and density of CNT/$CoSe_2$/C electrode.

Table S3. Comparison of density and volumetric capacity of various electrodes for SIBs.

Electrode material	Volumetric capacity (mA/cm^3)	Density (g/cm^3)	Ref.
**CNT/$CoSe_2$/C sponge**	**185.2**	**0.46**	**This work**
Sb@C microspheres	63	0.16	[8]
Sb/C fiber	65	0.16	[9]
Sb@nanoporous C	69	0.16	[10]
Sb/graphene	72	0.16	[11]
Sb/C ball milled	111	0.26	[12]
Yolk-shell Sb@C	186	0.35	[13]
Sb@C yolk-shell sphere	219	0.4	[14]
3D CNT/$MoSe_2$/C	267	0.93	[3]
Nanoporous Sb/C	421	0.98	[15]
Fe_2TeO_6	574.9	6.24	[16]

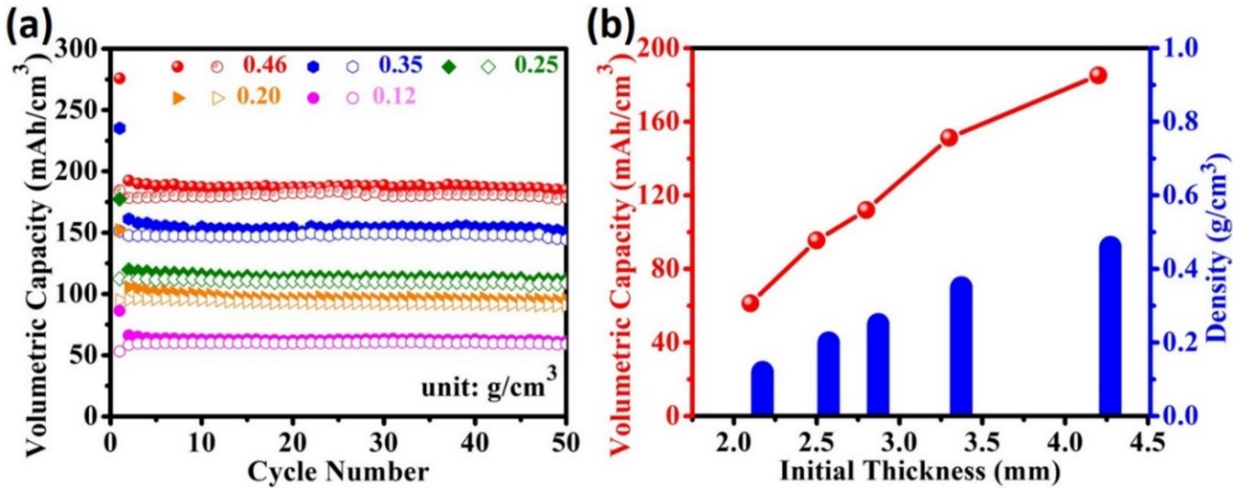

Figure S9. (a) The volumetric capacity of CNT/$CoSe_2$/C anode with various bulk density of electrodes at 100 mA/g. (b) The relationship among thickness, volumetric capacity and bulk density of CNT/$CoSe_2$/C anode.

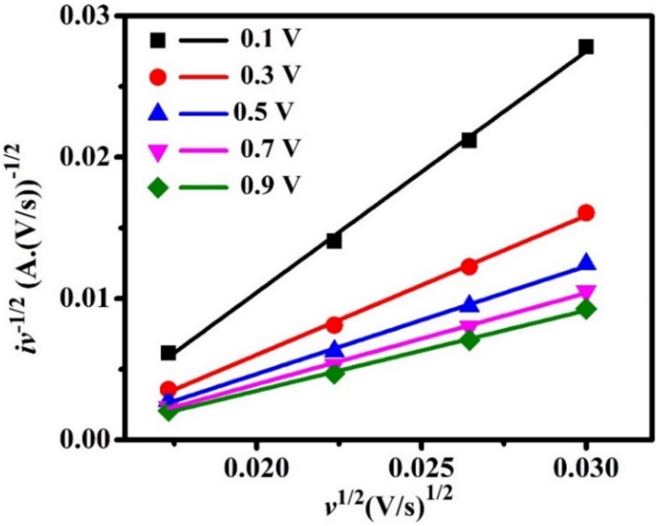

Figure S10. The plot of ($iv^{-1/2}$) *vs.* ($v^{1/2}$) at a fixed scan rate.

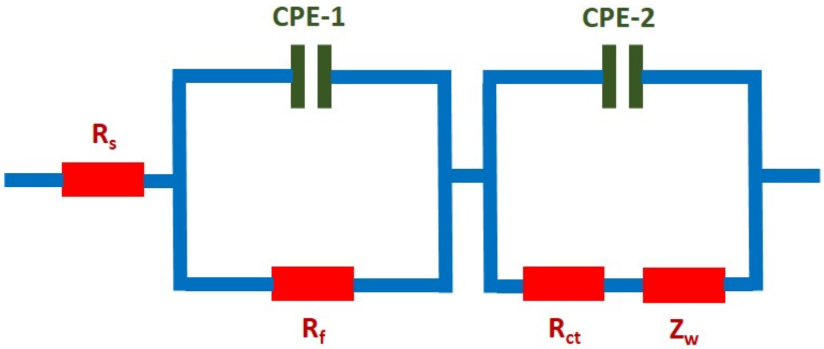

Figure S11. Equivalent circuit model to fit the EIS data.

Table S4. The values of R_s, R_{ct}, σ_ω and sodium diffusion coefficients D_{Na} in various electrodes after 30 cycle

Electrode Materials	R_s (Ω)	R_{ct} (Ω)	σ_ω	D_{Na} ($cm^2\ s^{-1}$)
CNT/$CoSe_2$	13.11	220.88	407.8	8.33×10^{-11}
CNT/$CoSe_2$/C	8.99	164.85	245.3	2.3×10^{-10}

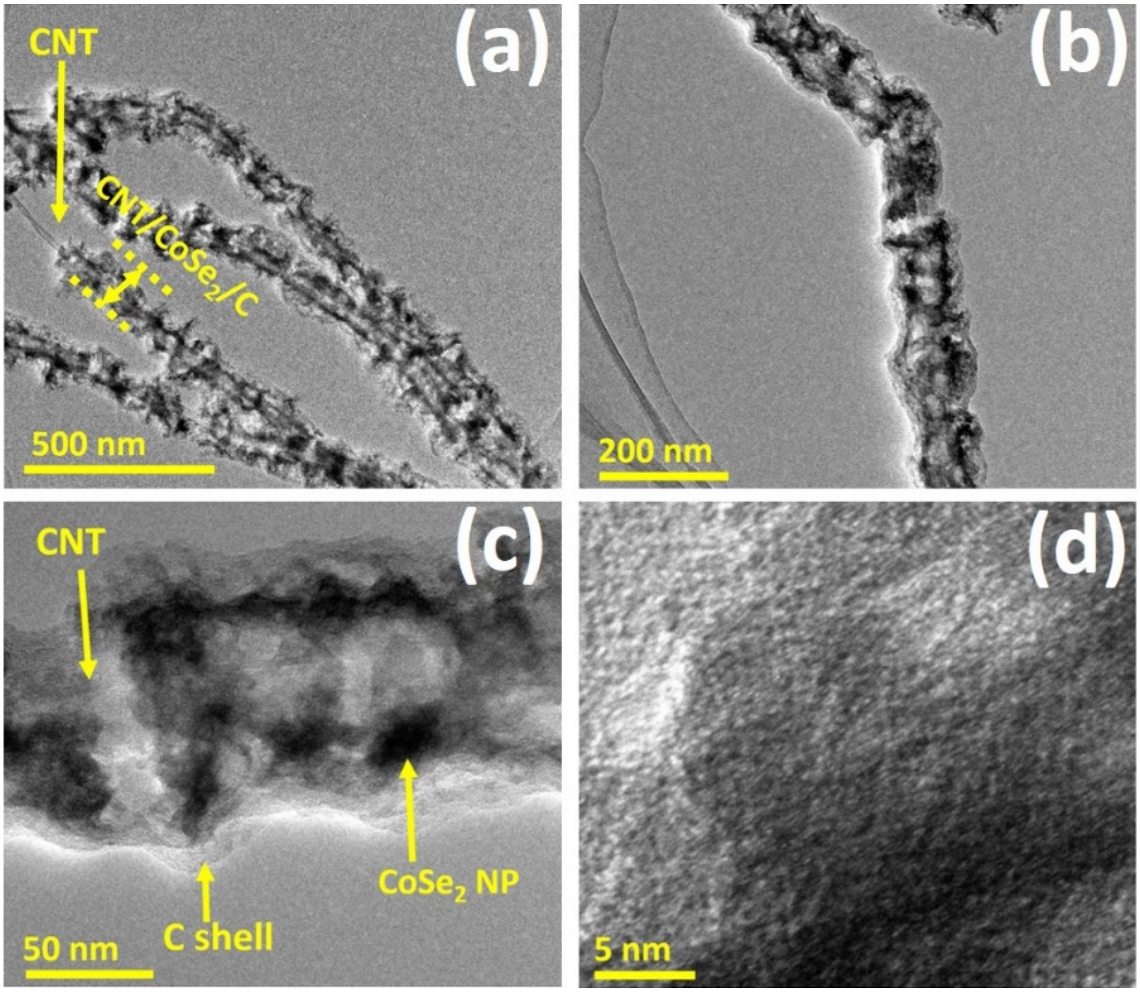

Figure S12. Ex-situ morphological analysis of CNT/$CoSe_2$/C after 100 cycles: (a, b, c) TEM micrographs showing its structural integrity, (d) HRTEM image of CNT/$CoSe_2$/C.

References

[1] X. Gui, J. Wei, K. Wang, A. Cao, H. Zhu, Y. Jia, Q. Shu, D. Wu, *Adv. Mater.* **2010**, 22, 617.
[2] Y. Chen, Y. Wang, Z. Wang, M. Zou, H. Zhang, W. Zhao, M. Yousaf, L. Yang, A. Cao, R. P. S. Han, *Adv. Energy Mater.* **2018**, 8, 1702981.
[3] M. Yousaf, Y. Wang, Y. Chen, Z. Wang, A. Firdous, Z. Ali, N. Mahmood, R. Zou, S. Guo, R. P. S. Han, *Adv. Energy Mater.* **2019**, DOI: 10.1002/aenm.2019005671900567.

[4] Y. Wang, Z. Wang, Y. Chen, H. Zhang, M. Yousaf, H. Wu, M. Zou, A. Cao, R. P. S. Han, *Adv. Mater.* **2018**, 30, e1802074.
[5] H.-Y. Chen, N. Bucher, S. Hartung, L. Li, J. Friedl, H.-P. Liou, C.-L. Sun, U. Stimming, M. Srinivasan, *Adv. Mater. Interfaces* **2016**, 3, 1600357.
[6] A. P. Vijaya Kumar Saroja, M. Muruganathan, K. Muthusamy, H. Mizuta, R. Sundara, *Nano Lett.* **2018**, 18, 5688.
[7] L. Suo, J. Zhu, X. Shen, Y. Wang, X. Han, Z. Chen, Y. Li, Y. Liu, D. Wang, Y. Ma, *Carbon* **2019**, 151, 1.
[8] Y. N. Ko, Y. C. Kang, *Chem. Commun. (Camb)* **2014**, 50, 12322.
[9] Y. Zhu, X. Han, Y. Xu, Y. Liu, S. Zheng, K. Xu, L. Hu, C. Wang, *ACS Nano* **2013**, 7, 6378.
[10] Q. Yang, J. Zhou, G. Zhang, C. Guo, M. Li, Y. Zhu, Y. Qian, *J. Mater. Chem. A* **2017**, 5, 12144.
[11] L. Hu, X. Zhu, Y. Du, Y. Li, X. Zhou, J. Bao, *Chemistry of Mater.* **2015**, 27, 8138.
[12] J. Qian, Y. Chen, L. Wu, Y. Cao, X. Ai, H. Yang, *Chem. Commun. (Camb)* **2012**, 48, 7070.
[13] J. Song, P. Yan, L. Luo, X. Qi, X. Rong, J. Zheng, B. Xiao, S. Feng, C. Wang, Y.-S. Hu, Y. Lin, V. L. Sprenkle, X. Li, *Nano Energy* **2017**, 40, 504.
[14] J. Liu, L. Yu, C. Wu, Y. Wen, K. Yin, F. K. Chiang, R. Hu, J. Liu, L. Sun, L. Gu, J. Maier, Y. Yu, M. Zhu, *Nano Lett.* **2017**, 17, 2034.
[15] B. Selvaraj, S.-S. Huang, C.-E. Wu, Y.-H. Lin, C.-C. Wang, Y.-F. Song, M.-L. Lu, H.-S. Sheu, N.-L. Wu, *ACS Appl. Energy Mater.* **2018**, 1, 2317.
[16] B. Shang, Q. Peng, X. Jiao, G. Xi, X. Hu, *Mater. Lett.* **2019**, 246, 157.
